# Pregnancy and Fetal Development: Cephalic Presentation and Other Descriptive Ultrasonographic Findings from Clinically Healthy Bottlenose Dolphins (*Tursiops truncatus*) under Human Care

**DOI:** 10.3390/ani10050908

**Published:** 2020-05-24

**Authors:** Pietro Saviano, Letizia Fiorucci, Francesco Grande, Roberto Macrelli, Alessandro Troisi, Angela Polisca, Riccardo Orlandi

**Affiliations:** 1 Ambulatorio Veterinario Saviano-Larocca, 41042 Spezzano, Italy; drpietro@hotmail.it (P.S.); fragrande@alice.it (F.G.); 2 Facultad de Veterinaria, Universidad de Las Palmas de Gran Canaria, Arucas, 35416 Las Palmas de Gran Canaria, Spain; 3 Dipartimento di Scienze Pure e Applicate, Università di Urbino, 61029 Urbino, Italy; macroberto@libero.it; 4 Scuola di Bioscienze e Medicina Veterinaria, Università di Camerino, 62024 Matelica, Italy; alessandro.troisi@unicam.it; 5 Dipartimento di Medicina Veterinaria, Università di Perugia, 06124 Perugia, Italy; angela.polisca@unipg.it; 6 Tyrus Veterinary Clinic, 05100 Terni, Italy; riccardo.orlandi83@hotmail.it

**Keywords:** ultrasonography, bottlenose dolphins (*Tursiops truncatus*), normal appearance, pregnancy, fetal position, fetal development, fetal welfare

## Abstract

**Simple Summary:**

Ultrasound data are vital for monitoring and detecting problems in pregnancies, and although there is a significant amount of data for domestic species, data for marine mammals are scarce. In domestic species, the use of ultrasonography to monitor a pregnancy usually has the following aims: fetal movements, fetal heart rates, measurements of the skull and the thorax for the prediction of the birth date interval, the morphological aspects of the fetal organs, the appearance of the umbilical cord, and the placentation. The purpose of this study is to provide to the clinician additional relevant data on fetal development and well-being during a dolphin pregnancy that may also be useful for wild population monitoring. This study is the result of a retrospective analysis of 192 ultrasound scans over 10 years that, for the first time, describes the sonographic findings of the bottlenose dolphin organogenesis and their correlation with the stage of pregnancy, as well as the calf presentation at birth, according to its position within the uterus, and moreover a complete literature review.

**Abstract:**

Ultrasonography is widely used in veterinary medicine for the diagnosis of pregnancy, and can also be used to monitor abnormal pregnancies, embryonic resorption, or fetal abortion. Ultrasonography plays an important role in modern-day cetacean preventative medicine because it is a non-invasive technique, it is safe for both patient and operator, and it can be performed routinely using trained responses that enable medical procedures. Reproductive success is an important aspect of dolphin population health, as it is an indicator of the future trajectory of the population. The aim of this study is to provide additional relevant data on feto-maternal ultrasonographic monitoring in bottlenose dolphin (*Tursiops truncatus*) species, for both the clinicians and for in situ population studies. From 2009 to 2019, serial ultrasonographic exams of 11 healthy bottlenose dolphin females kept under human care were evaluated over the course of 16 pregnancies. A total of 192 ultrasound exams were included in the study. For the first time, the sonographic findings of the bottlenose dolphin organogenesis and their correlation with the stage of pregnancy are described. Furthermore, this is the first report that forecasts the cephalic presentation of the calf at birth, according to its position within the uterus.

## 1. Introduction

A preventative medicine program is one of the key factors in health evaluation for ensuring the welfare of dolphins under human care. Ultrasonography (US) plays an important role in modern-day cetacean preventative medicine because it is a non-invasive technique, it is safe for both patient and operator, and it can be performed routinely using trained responses that enable medical procedures [1,2]. Ultrasound data are vital for monitoring and detecting problems in pregnancies, and while there is a significant amount of data for domestic species [3,4,5,6,7,8,9,10,11], data for marine mammals are scarce [12,13,14]. In domestic species, US is often used for the early identification of fetal pathologies and reabsorption, for example, altered/slowed down growth, loss of fetal fluids with decrease in the size of the vesicle and alteration of its shape, absence of heartbeat, blurring of margins and alteration of normal fetal anatomy, and detachment of the placenta from the uterine wall [3,4,5,6,7,8,9,10,11]. In marine mammal medicine, in the past, US was used only to confirm a pregnancy; however, an increasing number of facilities are now monitoring gestation by US, and further studies are now emerging with additional reference ranges.

Reproductive success is an important aspect of dolphin population health, as it is an indicator of the future trajectory of the population [15,16]. Pregnancy determination for wild dolphins, including differentiation of pregnancy stage, is possible during capture–release health assessments through application of diagnostic ultrasound to evaluate fetal development and viability, estimate gestational age, and measure anatomical structures [15,16]. The use of ultrasound for systematic pregnancy determination provides a useful tool for measuring an important component of reproductive success. Application of this approach for conservation of wild populations benefits from the establishment of baseline values, such as the estimates provided herein for the reference population of bottlenose dolphins [15,16,17,18,19,20,21,22].

The brightness (B)-mode technique is based on a process in which focused beams are iteratively sent into the body and the received waves are used to form an image scan-line, covering line-by-line the region of interest. The use of it to monitor bottlenose dolphin pregnancy dates back to the early 1990s, when Williamson et al. (1990) diagnosed pregnancy in a limited number of subjects (*n* = 4), at approximately the fourth month of gestation, being able to visualize fetal movements and fluids. Periodic monitoring allowed the authors to observe fetal vitality through the observation of cardiac mechanics and to carry out measurements both of the cranial diameter (on the front–occipital axis) and of the thoracic diameter, obtaining linear growth diagrams [23]. The authors showed difficulties in obtaining clear ultrasonographic images, both because the dolphins were uncooperative during the exam due to scarce training, and due to the features of the transducers used at the time. As regards the positioning of the transducer in the first months of gestation, the midline between the genital opening and the navel was used as a landmark, obtaining images in cross section, whereas in late pregnancy the probe was placed longitudinally, at 10–20 cm from the ventral midline [23]. Stone et al. (1999) observed a similar pattern of linear growth in bottlenose dolphins by measuring bi-parietal and thoracic fetal diameters from week 46 up to 1 week after delivery [24].

Lacave in her work developed an easy-to-use computer program to provide better birth prediction for regularly scanned dolphins during their gestation and to predict dolphin delivery dates, even with only one or two ultrasound scans of their animals [25]. Measurement of bi-parietal diameters is only possible when the head is distinguishable from the rest of the body; the head is presented ultrasonographically as a symmetrical ovoid structure and the bi-parietal diameters are measured where they reach their maximum amplitude [25]. For thoracic diameters, Lacave et al. (2004) used, as a reference point, the section where all cardiac chambers, symmetrically surrounded by the lungs, appear in the ultrasound image in the same section and where pectoral fins are also frequently visible. Lacave showed how the diameters of the skull increase more slowly than the thoracic diameters, and that the thoracic diameters represent the limit of accuracy in late pregnancy [25].

Sklansky et al. (2010) recognize the utility of fetal echocardiography as a safe technique able to evaluate the cardiovascular system in the bottlenose dolphin, especially in the period between the eighth and the ninth month of gestation. As in humans, this technique allows us to identify congenital cardiac anomalies [26,27,28] and to identify the possible causes of perinatal mortality risk associated with physiological abnormalities and cardiac hemodynamics [29,30]. In most cases, the optimal visualization of the fetal heart is obtained by positioning the pregnant female in lateral decubitus, homolaterally to the uterine horn in which the fetus is housed [26,27]. The optimal window is located near the maternal navel, proximal to the dorsal and caudal–ventral fin compared to the caudal fin. As expected, the cardiac dimensions increased with the approaching birth; passing from 3 to 6 cm of the 9th month up to 8–9 cm of the 10th month [26,27].

Recently, Ivancic et al. (2020) developed a protocol for feto-maternal ultrasonographic monitoring in bottlenose dolphins. In their work, a total of 203 US exams were performed during a 7-year period to monitor 16 pregnancies. The authors reported normal measurements and descriptive findings correlated with a positive outcome—fetal bi-parietal diameter, thoracic width in dorsal and transverse planes, thoracic height in a sagittal plane, aortic diameter, and blubber thickness all demonstrated a high correlation with date of gestation [21].

Umbilical cord accidents were diagnosed in the same dolphin in three consecutive pregnancies in a study by García-Párraga et al. (2014). The trans-abdominal ultrasound evaluation revealed the presence of a wrap of the umbilical cord around the fetal peduncle. All pregnancies ended in in utero death of fetuses and their expulsion [31]. In addition, an omphalocele (an abdominal wall defect at the base of the umbilical cord) in an approximately 16-week-old fetus was detected in the clinical case reported by Smith et al. (2013), thanks to the US prenatal examination. Color Doppler was utilized to study the blood flow within the omphalocele, as well as diagnose an associated anomaly of the umbilical cord, which contained three vessels instead of four [32]. Finally, the case of meconium aspiration syndrome (MAS) in a male neonate of bottlenose dolphin who died immediately after birth was reported by Tanaka et al. in 2014. At necropsy, a knot was found in the umbilical cord [33]. The lungs showed diffuse intra-alveolar edema, hyperemic congestion, and atelectasis due to meconium aspiration with mild inflammatory cell infiltration. Although the exact cause of MAS in this case was unknown, fetal hypoxia due possibly to the umbilical knot might have been associated with MAS, which is the first report in dolphins. MAS due to perinatal asphyxia should be taken into account as a possible cause of neonatal mortality and stillbirth of dolphin calves [33].

Valuable information about the ontogeny of the body systems and their development in cetacean species, the precise time intervals of such developments, and any distinctive growth trajectories are basically unknown, because descriptions are based on occasional recoveries of embryos and fetuses and it very difficult to acquire complete ontogenic series [34,35,36,37,38,39]. Fetal abnormalities have been observed in cetaceans, as in other species. Brook, in 1994, for the first time, described the ultrasound diagnosis of an anencephaly, a lethal form of cephalic axial skeletal-neuronal disraphism, in a *Tursiops aduncus* fetus [34]. The fetal skull base appeared disproportionately small, and the cranium could not be identified. Fetal heart motion could be detected throughout the gestation. After a period of 357 days after conception, an uncomplicated, spontaneous delivery produced a stillborn male anencephalic calf. The abnormality was associated with various factors including respiratory tract infection in early gestation and folic acid deficiency. This case illustrates the ability of US to provide assessment of fetal morphology and growth, information not available by other means [34]. Ultrasonography proved to be appropriate to monitor fetal development, placenta and fetal membranes, and also to identify umbilical cord defects, thanks to the monitoring of its position and perfusion. This method has the advantage that it can identify abnormalities at early gestational stages [21,34], and that regular measurements allow monitoring of fetal growth and provide a more accurate prediction of expected delivery [25], so that adequate arrangements can be made in good time. The aim of this retrospective study was to provide additional relevant descriptive findings on feto-maternal ultrasonographic monitoring protocols in bottlenose dolphin species, thanks to the analysis of 192 ultrasound exams during a 10-year period. The data obtained may be very useful for the future clinical practice for managed population and in situ population studies, as it can be used to improve the understanding of the pathophysiology of reproductive failure.

## 2. Materials and Methods

### 2.1. Study Animals

This study is the result of a retrospective analysis of 192 ultrasound scans obtained during the routine pregnancy check of bottlenose dolphins and from the marine mammal ultrasound consulting work in 11 different facilities over 10 years. All the examinations were included in the preventative medicine protocol of each facility, and no additional examinations were performed. From 2009 to 2019, serial ultrasonographic exams of 11 healthy bottlenose dolphin females (average age: 18 ± 7 years; min–max: 9 to 36 years) kept under human care were evaluated over the course of 16 pregnancies. Three dams were pluriparous. The calves born were 10 males and 6 females; of these, 2 females died 9 days after birth due to a respiratory disease. Neither case was that of cephalic birth. Inclusion criteria involved all pregnancies ended with the birth of alive calves that survived at least 48 h after the delivery. Exclusion criteria included any pre-existing health conditions that could have affected pregnancy, abortion, or any significant health conditions that occurred during pregnancy and required initiation of treatment by the attending clinician. A total of 192 ultrasound exams were included in the study. All examined animals were trained routinely for medical behaviors, including US. Lateral and ventral abdominal scanning was performed using seawater for acoustic coupling. For the study, the urogenital area was explored and the reproductive organs, ovaries, and uterus were evaluated. Uterine fluid echogenicity was assessed as anechoic, hypo-echoic, or hyper-echoic, as well as for the presence of echoic free-floating particles. Umbilical cord vasculature was assessed in cross section. Color Doppler confirmed vascular flow.

### 2.2. Ultrasonography: Instrumentation and Methodology

A portable SonoSite 180 Plus with a 2–5 MHz convex probe, an Esaote Mylab 25 gold with convex probe 2–5 MHz, an Aloka 900 with convex probe 2–5 MHz, and a General Electrics Logiq V2 with a 2–5 MHz curvilinear transducer were used to evaluate the dolphins during pregnancy. During the exam, the machine was covered with a transparent plastic bag to avoid accidental contact of the device with salt water. To avoid direct sunlight, a dark-colored bag was used to cover the instrument. The probe was waterproof. Acoustic gel was unnecessary because water provides an excellent medium through which to conduct ultrasound waves. Still images obtained were stored in DICOM (Digital Imaging and Communications in Medicine) format, whereas videos were also recorded using an external hard-disk.

### 2.3. Statistical Analyses

For each fetus, the time required for the appearance of each organ studied was analyzed. The quantitative variable “organ onset time” was introduced and a descriptive statistic expressed—average organ onset time with relative standard deviation. In addition, left or right ovulation rates and podalic or cephalic birth rates were analyzed. Medcalc, version 11.6.0.0, was used to analyze the data.

## 3. Results

**Figure 1:**
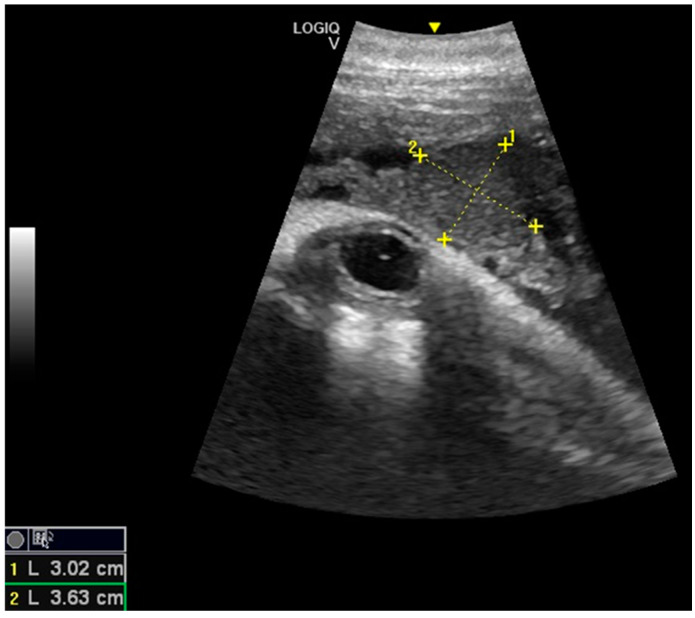
Corpus luteum (CL) with measurements.

Considering the 16 pregnancies, the percentage of ovulation in the left ovary was 68.75%, whereas the ovulation in the right ovary was 31.25%, and it was of interest that two dams always ovulated in the right ovary. Maximum corpus luteum (CL) longitudinal diameter was 3.63 cm and transversal diameter was 3.02 cm (Figure 1), even though the diameter may vary according to laterality. The results concerning the gonadal activity correspond to previous studies [18,19,20].

**Figure 2:**
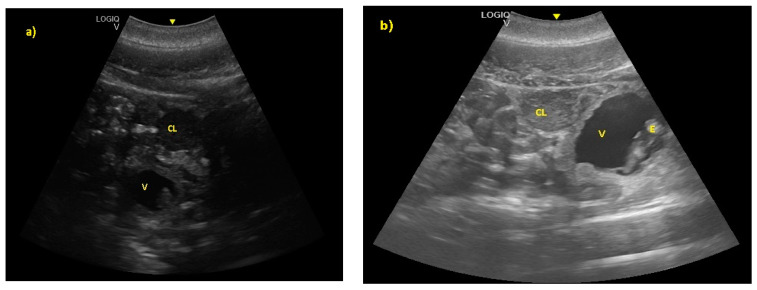
(**a**) The embryonic vesicle at 38 ± 2 days post-ovulation appeared as a roundish structure with an anechoic content and a hyper-echoic structure inside (V), under the CL. (**b**) At 52 ± 3 days, the embryo was perfectly recognizable (E).

The embryonic vesicle was recognizable at 29 ± 3 days post-ovulation on the apex of the uterine horn as a roundish structure with an average diameter of 1.21 cm with an anechoic content. In the following week, it was possible to recognize the embryo inside it as an elongated hyperechoic structure (Figure 2).

**Figure 3:**
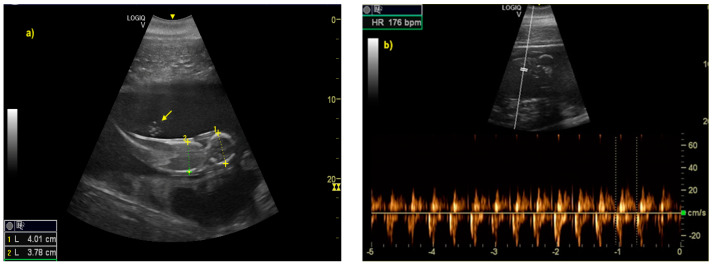
(**a**) The distinction between head and trunk was clear between 68 ± 5, up to the 216 ± 5 day of gestation. It allowed measurement of bi-parietal diameters. The umbilical cord was already easy guessed (as indicated by yellow arrow). (**b**) The fetal heart rate (HR) was measured once the cardiac mechanics became visible.

**Figure 4:**
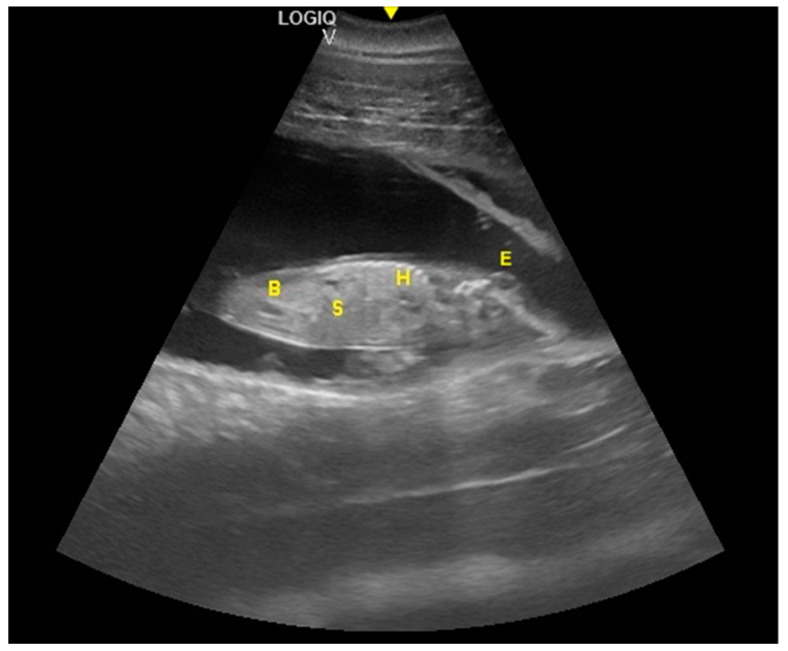
Fetal stomach (S) and fetal urinary bladder (B) were the first abdominal organs to be visualized, and appeared as distinct and anechoic cavities. The heart was recognizable as an anechoic cavity (H) and the embryonic cardiac mechanics were displayed as a point of maximum fluctuation of the echoes. The eye appeared as an anechoic cavitary structure (E).

The distinction between head and trunk was visible starting from 68 ± 5 days after ovulation. From the 216 ± 5 days of gestation, measurements started to be hard to realize with accuracy. In fact, in the evaluations after this last period, the position/orientation of the fetus and its size meant that it was not possible to take reliable measurements thereafter. Starting from 68 ± 5 days after ovulation, the embryonic cardiac mechanisms were displayed as a point of maximum fluctuation of the echoes. The heart rate was measured because the cardiac mechanics became visible and remained constant between 155 and 198 bpm until the ninth month of pregnancy (Figure 3). During the last 3 months, it stabilized at 140 bpm, to reach 85 ± 5 bpm in the last 2 weeks of gestation. The first abdominal organs to be visualized were the stomach and the urinary bladder (98 ± 3 and 110 ± 2 days of gestation, respectively), which appeared as distinct and anechoic cavities. It was also possible to recognize the eye as an anechoic cavitary structure (Figure 4).

**Figure 5:**
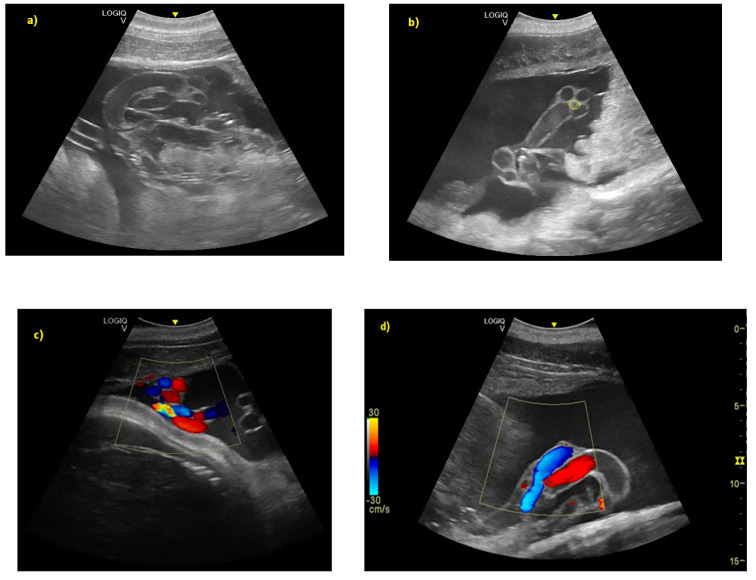
(**a**) From day 119 ± 6 of gestation, the umbilical cord was clear as a hyper-echoic cordoniform structure. (**b**) The small hypo-echoic central cavity is the urachus, shown by the yellow circle. (**c**) Color Doppler demonstrating flow within the umbilical vasculature: (**d**) the umbilical veins (in blue), and the umbilical arteries (in red).

**Figure 6:**
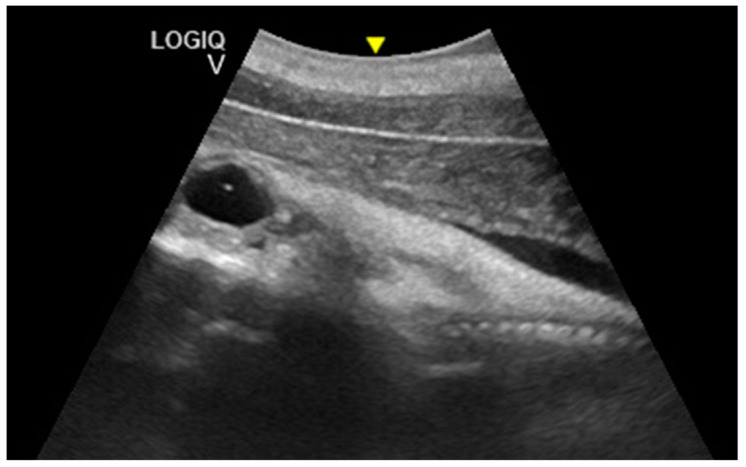
From day 110 ± 2 of gestation, the eye appeared as anechoic cavitary structures, whereas starting from the 149th day, the lens was also visible. At 167 ± 3 days of gestation, it was possible to identify the teeth.

The distinction between thorax and abdomen and, thus, the presence of the diaphragm, was seen at 92 ± 5 days of gestation. A clear distinction between lungs and liver was identified at 112 ± 5 days, whereas the ribs were visible at 153 ± 5 days. At 167 ± 3 days of gestation, the dorsal fin was visible as a hyper-echoic triangular structure in the dorsal portion of the trunk. During the same period, it was possible to identify the teeth. Even if the umbilical cord was easily guessed previously (as shown in Figure 3) from the 119 ± 6 day of gestation, it was clear as a hyper-echoic cordoniform structure, and it was important to evaluate the internal vascular components and the absence of knots or torsions until the birth (Figure 5). Furthermore, it was possible to notice, between the 149th day and the 230th day of gestation, that the eye was open, the lens was visible, and eyelid movements were also detectable (Figure 6).

**Figure 7:**
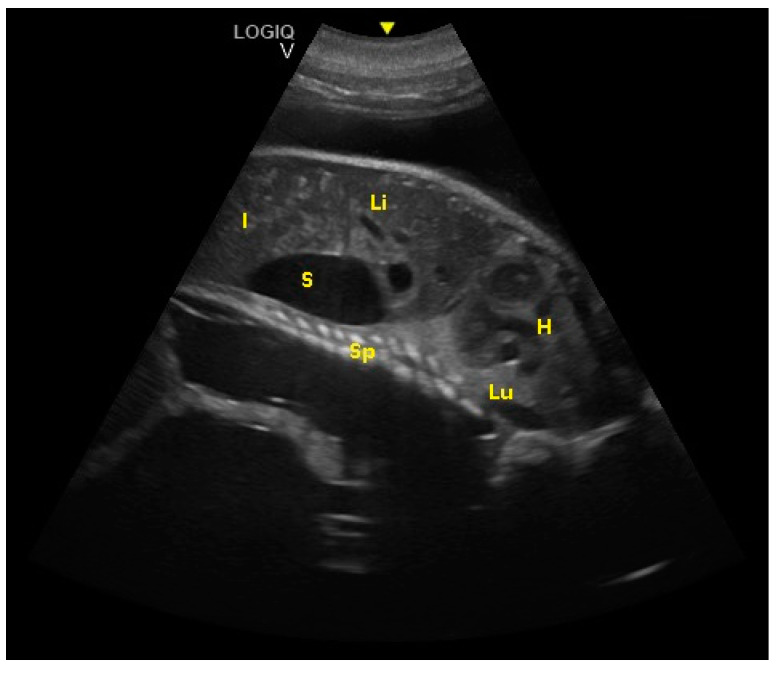
Fetal spinal cord (Sp), stomach (S), liver (Li), intestine (I), lungs (Lu), and heart (H) were visualized at 194 ± 5 days of gestation.

The intestine was visualized at 189 ± 5 days of gestation. The cardiac chambers were visualized 194 ± 5 days after ovulation, and after about 3 weeks, the vascular structures (aorta and caudal vena cava) departing from them were visualized (Figure 7).

**Figure 8:**
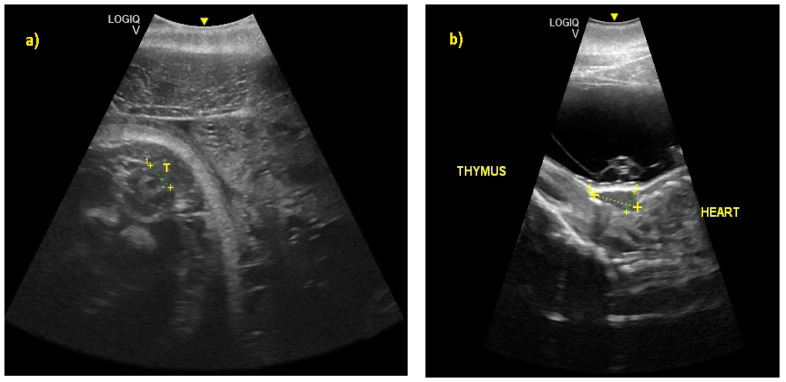
(**a**) At 245 ± 2 to 288 ± 2 days of gestation, it was possible to recognize the thyroid (T) and (**b**) the thymus, respectively.

From the 230th day of gestation, it was possible to observe a ventral flexion of the caudal fin, a hyper-echoic structure, in contact with the abdomen. From the 245th to the 288th day of gestation, it was possible to recognize the thyroid and thymus (Figure 8). During the last 3 months of gestation, it was possible to identify the kidneys.

**Figure 9:**
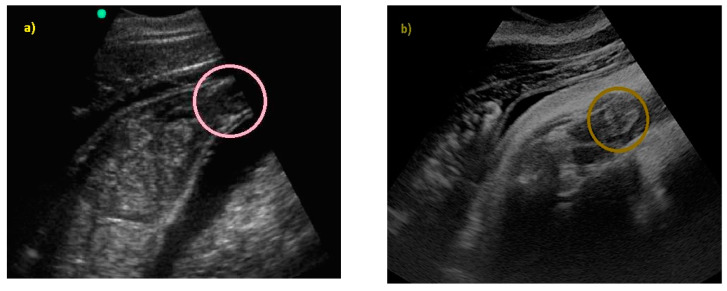
(**a**) An ultrasound image of female genitals (apricot-shaped) by SonoSite 180 Plus with a 2–5 MHz convex probe; (**b**) the same area caught with General Electrics Logiq V2, with a 2–5 MHz convex probe.

In addition, it was possible to identify the genitalia and sex the fetus (males have a tri-lobed structure, whereas females have an apricot-shaped structure, as shown in Figure 9). 

**Figure 10:**
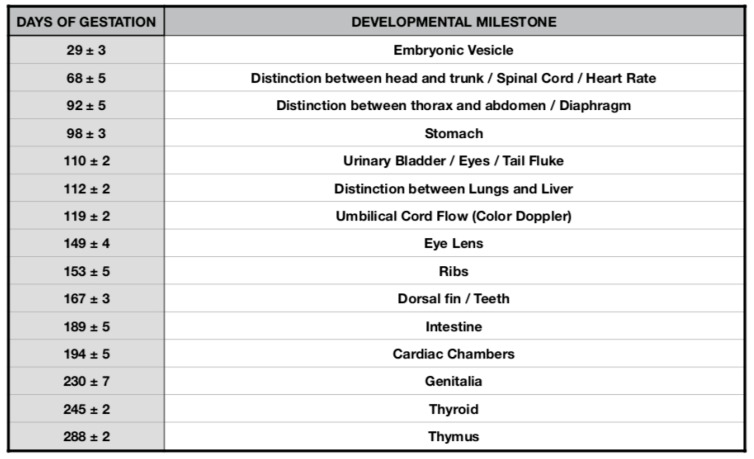
Bottlenose dolphin (*Tursiops truncatus*) organogenesis timeline table.

Starting from the 301th post-ovulation day until the end of pregnancy, it is very difficult to obtain images of the caudal portions of the fetus, due to the folded position it assumes within the maternal uterus. To the authors’ knowledge, this is the first study that reports the sonographic descriptive findings of the bottlenose dolphin organogenesis and their correlation with the stage of pregnancy as shown in Figure 10).

**Figure 11:**
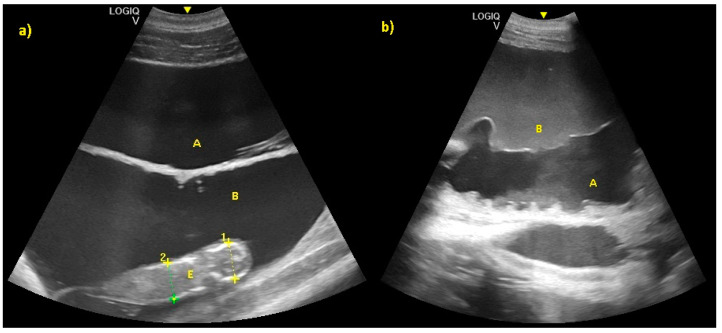
(**a**) Allantoic (A) and amniotic fluid (B) in early stage of pregnancy. (**b**) Allantoic fluid appears as an anechoic fluid (A), and the amniotic fluid appears as a hyper-echoic fluid, with an increasing amount of echoic particles during the last period of pregnancy (B).

Allantoic and amniotic fluid are distinguishable in all examinations starting from the last trimester of pregnancy. Allantoic fluid appears as an anechoic fluid and the amniotic fluid appears as a hyper-echoic fluid, with an increasing number of echoic particles during the last period of pregnancy (Figure 11).

**Figure 12:**
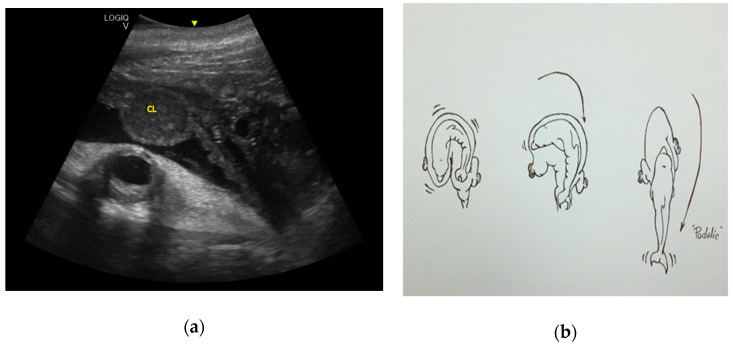
(**a**) Using the CL as a reference, if the fetus skull is located close to the CL, it will have a podalic position at birth, (**b**) as shown in the image.

Finally, fetal position was evaluated throughout the gestational period. Considering the 16 pregnancies, the percentage of fluke presentation was 93.75%, whereas the head presentation was 6.25%. It is interesting to note that they were all successful cephalic deliveries. According to the results of the present study, it is possible to predict the calf presentation at birth, considering its position in the uterus during the last trimester. Using the CL as a reference, if the fetus skull is located close to the CL, it will have a podalic position at birth (Figure 12).

**Figure 13:**
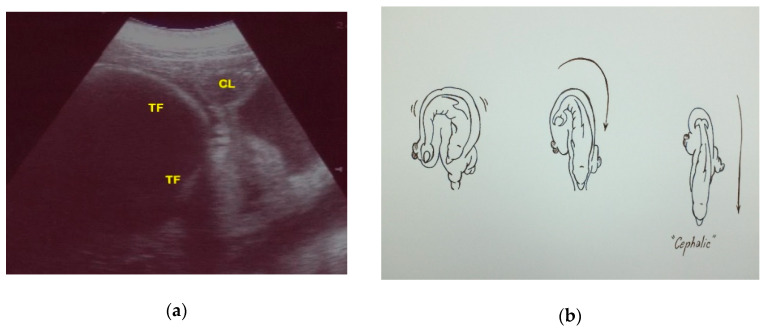
(**a**) Using the CL as a reference, if the tail fluke (TF) is located close to the CL, it will have a cephalic position at birth, (**b**) as shown in the image.

However, if the tail fluke is located close to the CL, it will have a cephalic position at birth (Figure 13).

To the authors’ knowledge, the present study reports the first bottlenose dolphin cephalic presentation documented by US.

## 4. Discussion

In the present retrospective study, we describe, by US, the genesis of the fetal organs (stomach, bladder, lungs, eye, intestine) and structures (column, appendices), and note that their appearance can be used to estimate the gestational period in the dolphin, in the absence of further information, as described in other species [40]. To the authors’ knowledge, this is the first study that reports the sonographic descriptive findings of bottlenose dolphin organogenesis and their correlation with the stage of pregnancy. The heart, while appearing visible already in the early stages of gestation, was displayed optimally between the eighth and the ninth month, allowing exclusion of the presence of detectable pathologies. As reported by Sedmera et al. (2003), there is a scant amount of data regarding heart development in cetaceans [41]. In their study, the authors examined samples from a unique collection of embryonic dolphin specimens macroscopically and histologically to learn more about normal cardiac development in the spotted dolphin. It was found that during the spotted dolphin’s 280 days of gestation, the heart completes septation at about 35 days. However, substantial trabecular compaction, which normally occurs in terrestrial mammals, as well as in humans at around the same time period, was delayed until day 60, when coronary circulation became established. By day 80, however, the heart gained a compacted, characteristic shape, with a single apex [41]. Considering the bottlenose dolphin’s 385 days of gestation, around 68 ± 5 days after ovulation, the results of the present study show how the heart was recognizable by US, and the embryonic cardiac mechanics were displayed as a point of maximum fluctuation of the echoes. An accurate index of fetus welfare is the fetal heart rate [26,27,28,29,30]. The heart rate was measured once the cardiac mechanics became visible, and remained constant between 155 and 198 bpm until the ninth month of pregnancy. During the last 3 months, it stabilized at 140 bpm, to reach 85 ± 5 bpm in the last 2 weeks of gestation.

The distinction between thorax and abdomen and, thus, the presence of the diaphragm, was seen between 92 ± 5 days of gestation, whereas the clear distinction between lungs and liver was identified at 112 ± 5 days of gestation. The correlation between the dolphin fetus organogenesis and the gestational period may help the clinician to identify the proximity of the delivery and any suspected anomaly in fetal development. In human literature, normal fetal lung and lung/liver echogenicity relationships facilitate early diagnosis of congenital bronchopulmonary abnormalities, and a decrease in the fetal lung/liver echogenicity has been shown to predict respiratory distress in newborns [21]. Regarding the two calves who died 9 days after birth due to a respiratory disease, there are no data that suggested lung alteration identified by ultrasound throughout the pregnancy. The two clinical cases had fetal development comparable with the other fetuses examined. The necropsy of both calves revealed postpartum pathological process as cause of death.

The umbilical cord has also been documented in ultrasound images, thus being able to exclude the presence of twisting nodes or echographically detectable defects of the same conditions that proved fatal in the bottlenose dolphins [31,32,33]. Color Doppler may be utilized to study the blood flow, as well as diagnose an associated anomaly of the umbilical cord, such as in the case of an onphalocele that contained three vessels instead of four [32]. It is worth mentioning that four vessels are needed as normal presentation, as shown in Figure 4.

As described in other animals such as horses [42], during the last 20 days of pregnancy, amniotic fluid shows an increase of the echogenicity compared with allantoic fluid. Allantoic fluid has to remain completely anechoic during all the stages of pregnancy. Amniotic and allantoic fluids increase their volume during pregnancy, giving protection to the fetus. At the time of birth, allantoic fluid dilates the pathways of birth, whereas amniotic fluid has the function of lubricating the fetus [43]. The allantoic liquid is composed mainly of the urine of the fetus and has an anechoic aspect throughout the pregnancy [43]. The amniotic fluid envelops the fetus, and epithelial cells and meconium are accumulated in it, causing a progressive increase of its echogenicity compared to the allantoic liquid [43]. As confirmed by the present study, the difference in echogenicity between these two liquids reaches the highest level in the last 20 days of pregnancy because of the increase in the number of echogenic particles in the amniotic fluid. The authors hypothesize that these observations could be used to determine the approximate date of birth. Echogenic particles may indicate pathology and fetal stress when associated with infective debris, however, increased fluid echogenicity is not always predictive of pathology [21]. In all pregnancies evaluated in the present study, no changes in the allantoic fluid were observed throughout the gestation period. The results of the present study agree with recently published data by Ivancic et al. (2020) and provide further support for the newly established reference ranges [21].

Fetal position is another aspect to evaluate throughout gestation. In fact, keeping both the CL and the fetus in the same scan, it is possible to evaluate how, during the last three months of pregnancy, the relative topographical position of the head to the CL of the fetus is related to a podalic presentation at birth, which is the normal condition in this species (93.75% of cases). On the contrary, when the tail of the fetus is topographically related to the CL in the same image, it is related to a cephalic birth (6.25%). It is known that a cetacean fetus can change its position in the uterus during pregnancy, but usually moves into a tail-first position by the last months [36,44]. Head presentation of a fetus is reported for several delphinid species, and is thought to be an adaptation to the pregnancy and delivery in the aquatic environment [19,44,45,46,47,48,49,50,51,52,53]. It is repeatedly observed under human care—the rate of cephalic delivery was found to be 7% in killer whales (*Orcinus orca*) and 1.2% in bottlenose dolphins [19]. The labor of eight captive finless porpoises (*Neophocaena asiaeorientalis asiaeorientalis and Neophocaena asiaeorientalis sunameri*) were described by Deng et al. in 2019. The duration of parturition and the time of particular events in the parturition process were recorded for both podalic and cephalic births. Cephalic births were shorter than podalic births, and the calf position at birth did not seem to have a negative effect on its survival [47]. Successful cephalic delivery in a bottlenose dolphin under human care is described in detail by Essapian (1963)—stage 2 of the parturition lasted 22 min [49]. The result corresponds to that of the present study; in fact, the cephalic birth mentioned here also had a duration of 22 min. Even if it is indicated as a complicating factor, such cases are not referred to as pathologies [49]. In the wild, head presentation of a fetus was observed in a stranded dead white-beaked dolphin (*Lagenorhynchus albirostris*) [50]; it had ruptured the uterine wall, and thus the head presentation could be a cause of both dystocia and maternal death [50]. Numerous cases of cephalic delivery have been consistently reported for belugas (*Delphinapterus leucas*) in both nature and under human care; a rate of head-first delivery in captive belugas of 14% has been reported [51,52,53]. Head presentation seems to be more widespread in belugas than in any cetacean species. The cephalic presentation of a fetus in cetaceans is not a pathology but a natural variation. This idea is also supported by the documented cases of successful cephalic deliveries and by the occurrence of head presentations both in the wild and under human care. Nonetheless, head presentation of a fetus in a small cetacean can increase the risk of trauma or dystocia. In addition, a change of presentation at an advanced stage can be a pathology, such as that described by Baker and Martin in 1992 [54]. To the authors’ knowledge, the present study reports the first bottlenose dolphin cephalic presentation documented by US. An accurate and early ultrasound diagnosis of the presentation at delivery can help the clinician to adapt the intervention protocol to the needs of the case and, thus, minimize the risk of reproductive failure. The results of the present study reinforce the importance of monitoring health and fetal vitality throughout the duration of pregnancy and at the time of delivery by ultrasound.

In spite of the advantages provided by ultrasonographic surveys of dolphins, the procedure has some limitations compared to the same examination carried out in species commonly investigated in clinical practice [1,2]:

(1) it must be conducted by an expert operator;
(2) it depends on the features of the device;
(3) animals must be trained for the voluntary medical behavior;
(4) the animals must remain in water, which may not be safe for the instrumentation;
(5) the external environment (and the light level, in particular) negatively affects results.

However, the difficulties listed above can be overcome, given the value of the information that can be gained, as the methods have been successfully applied to both under human care and wild dolphins.

## 5. Conclusions

This study adds further findings to the ultrasonographic monitoring protocol of bottlenose dolphin pregnancy. On the basis of the review of the literature, this is the first study that describes the sonographic data of bottlenose dolphin organogenesis and their correlation with the stage of pregnancy. As described in other species [40], these data could be used to estimate the gestational period in the dolphin in the absence of further information (such as measurements that allow derivation of linear growth diagrams, or a known ovulation date). These findings may be useful for investigations of stranding dolphins, providing data on prenatal development. These data are otherwise difficult to establish in wild cetaceans, as the precise time intervals of such developments and any distinctive growth trajectories in most cetacean species are basically unknown [34,35,36,37,38,39]. Furthermore, this is the first report that describes by ultrasonography the cephalic presentation of the calf at birth, according to its position within the uterus. The results of the present study reinforce the importance of monitoring health and fetal vitality throughout the duration of pregnancy and at the time of delivery by ultrasound. Reproductive success is vital in sustaining cetacean populations, and the systematic use of ultrasound for pregnancy monitoring provides a useful tool for assessing reproductive success, including for free-ranging dolphins. During capture–release health assessments, it is possible through application of diagnostic ultrasound to evaluate fetal development and viability, estimate gestational age, and measure anatomical structures [16]. This wild population conservation approach benefits from the findings of studies of the population of bottlenose dolphins under human care. Deviations from the normal findings during pregnancy could be related to the alteration of the health status and the well-being of the fetus or the mother, and, if detected sufficiently early, could result in timely therapeutic intervention for animals under human care. Thus, findings related to a reproductive failure in the wild may also be elucidated. However, further investigation will be necessary, carried out with a greater number of subjects, in order to validate the results obtained and to apply these diagnostic methods to other cetacean species.
